# The Perceived Impact of COVID-19 on the Mental Health Status of Adolescent and Young Adult Survivors of Childhood Cancer and the Development of a Knowledge Translation Tool to Support Their Information Needs

**DOI:** 10.3389/fpsyg.2022.867151

**Published:** 2022-05-30

**Authors:** Sharon H. J. Hou, Andrew Tran, Sara Cho, Caitlin Forbes, Victoria J. Forster, Mehak Stokoe, Elleine Allapitan, Claire E. Wakefield, Lori Wiener, Lauren C. Heathcote, Gisela Michel, Pandora Patterson, Kathleen Reynolds, Fiona S. M. Schulte

**Affiliations:** ^1^Department of Oncology, Division of Psychosocial Oncology, Cumming School of Medicine, University of Calgary, Calgary, AB, Canada; ^2^Department of Psychology, British Columbia Children’s Hospital, Vancouver, BC, Canada; ^3^The Hospital for Sick Children, Toronto, ON, Canada; ^4^School of Women’s and Children’s Health, UNSW Medicine and Health, UNSW Sydney, Sydney, NSW, Australia; ^5^Kids Cancer Centre, Sydney Children’s Hospital, Randwick, NSW, Australia; ^6^Center for Cancer Research, National Cancer Institute, NIH, Bethesda, MD, United States; ^7^Health Psychology Section, Department of Psychology, Institute of Psychiatry, Psychology and Neuroscience, King’s College London, London, United Kingdom; ^8^Department Health Sciences and Medicine, University of Lucerne, Lucerne, Switzerland; ^9^Research, Evaluation and Policy Unit, Canteen Australia, Sydney, NSW, Australia; ^10^Faculty of Medicine and Health, University of Sydney, Sydney, NSW, Australia; ^11^Long Term Survivor’s Clinic, Alberta Children’s Hospital, Calgary, AB, Canada; ^12^Department of Medicine, Faculty of Family Medicine, University of Calgary, Calgary, AB, Canada

**Keywords:** pediatric oncology, psychosocial oncology, adolescent and young adult cancer, survivors of childhood cancer, mental health, COVID-19

## Abstract

**Background:**

Adolescent and young adult (AYA; 13 to 39 years) survivors of childhood cancer may be especially vulnerable to physical health and mental health concerns during the pandemic. We investigated the impact of COVID-19 on the mental health status of AYA survivors (Aim 1) and shared tailored, evidence-based health-related information on COVID-19 (Aim 2).

**Methods:**

Between May and June 2020, participants completed a cross-sectional online survey assessing their cancer history, current mental health status, and their COVID-19 information needs.

**Results:**

Ninety-four participants (78 females, 13 males, 2 non-binary) with a mean age of 26.9 years (SD = 6.2) were included in the final sample. Participants reported residing from 10 countries and 94% identified as White. Nearly half of the participants (49%) described their mental health status as worse now than before the pandemic. Thirty-nine participants (41%) that indicated their current mental health status was tied to fears/worries about their past cancer and treatment experienced a higher level of anxiety and PTSS than those who did not report the same. Most participants (77%) had not received any information related to the potential risks of COVID-19 and expressed an interest in receiving this information. In response, an infographic detailing recommended strategies for coping with mental health problems in the pandemic, along with preliminary study findings, was developed.

**Discussion:**

AYA survivors reporting their mental health status was linked to their past cancer experienced poorer mental health. There is a value to educating survivors on their potential health risks, but accounting for their perceived mental health vulnerabilities should be considered when disseminating knowledge. The use of an infographic is a unique contribution towards the development of innovative and personalized means of sharing health education to this vulnerable yet resilient group. This research on the mental health status of AYA survivors very early in the pandemic informs continued initiatives investigating the rapidly changing nature of how COVID-19 may impact AYA survivors today and in the future.

## Introduction

Coronavirus disease 2019 (COVID-19) is an infectious disease caused by a severe acute respiratory syndrome coronavirus 2 (SARS-CoV2; World Health Organization, 2020). As of January 2022, there are over 364 million cases and 5.63 million deaths confirmed worldwide (WHO Coronavirus (COVID-19) Dashboard, 2021). Many individuals infected with COVID-19 experience a range of mild to moderate respiratory symptoms and can recover without medical intervention. Those with underlying medical comorbidities or immunocompromised individuals are among the most vulnerable populations that may be at a greater risk for developing serious illness or dying from COVID-19 (Coronavirus (COVID-19) and the Immunocompromised, 2021).

Due to advances in medical treatment, there is a growing prevalence of survivors of childhood cancer around the world. In fact, 5-year survival of children and youth with cancer is beyond 80% in most European and North American countries (Robison and Hudson, 2014). Further, there is an estimated >500,000 survivors of childhood cancer in North America (Robison and Hudson, 2014). The most common forms of childhood cancer include leukemias, brain cancers, and solid tumour (Steliarova-Foucher et al., 2017; Siegel et al., 2021) Survivors of childhood cancer may be especially susceptible to the impacts of COVID-19 (Forster and Schulte, 2021) due to chronic health conditions, known as late effects, that stem from their cancer treatment (Schulte et al., 2020). These include health concerns that are known to increase risk of a more severe course of COVID-19, including pre-existing cardiac issues, pulmonary disorders, obesity, and diabetes (Neville et al., 2006; Brabant et al., 2012; Ward et al., 2014). Psychological late effects, including anxiety, depression, and post-traumatic stress symptoms (PTSS), are also common in survivors of childhood cancer (Lown et al., 2015; Brinkman et al., 2018; Nathan et al., 2018), and predisposes these individuals to mental health vulnerabilities.

Adolescents and young adults (AYAs) survivors of childhood cancer (henceforth referred to as AYA survivors) have distinct needs. It can be challenging for AYA survivors to meet their health care needs and navigate survivorship (Barr et al., 2016) because this is a critical developmental period during which major changes take place in their living arrangements, health care needs, and transition from pediatric to adult care (Marjerrison and Barr, 2018). Prior to the COVID-19 pandemic, AYA survivors reported experiencing poorer quality of life, including physical and mental health, when compared to the general population (Schulte et al., 2021).

There is emerging research on the negative impact of COVID-19 on the mental health of the general population (Cullen et al., 2020; Pfefferbaum and North, 2020; Usher et al., 2020), as well as those from vulnerable groups (Nearchou et al., 2020). Several studies have explored the psychosocial effects of the COVID-19 on those living with and beyond their cancer, highlighting a greater prevalence of mental health concerns than individuals without a cancer history as well as an enduring sense of fear and worry about their potential health risks (Wang et al., 2020; Islam et al., 2021; Page et al., 2022). Of note, the pandemic has drastically changed the access to and model of delivery of follow-up care for survivors (replacing face-to-face appointments to virtual health), which has implications for the surveillance of their health and psychosocial needs (McLoone et al., 2020). Further, quarantine and social isolation (Pahl et al., 2021) may contribute to feelings of isolation and loneliness (e.g., Brinkman et al., 2018) that further compromise one’s mental health (e.g., Nearchou et al., 2020).

AYA survivors may be at a greater risk of mental health concerns given their health history and perceived vulnerabilities. Indeed, a mixed-methods study conducted in the United States at the outset of the pandemic found that AYA survivors reported a high level of anxiety regarding their health and that of their family, feelings of isolation, and worries about employment status (Shay et al., 2021). In another international study comprised of AYA patients and survivors, more than 50% of respondents expressed an interest for information tailored to their needs in coping with the pandemic (Košir et al., 2020). More recently, a study investigating sources of COVID-19 information use by AYA survivors with cancer living in Canada revealed a preference by AYA survivors to seek information through social media and websites of cancer organizations (Yan et al., 2021). Collectively, existing research highlights a need to support AYA survivors in their coping with their mental health and an urgency to deliver health information to this group, likely through innovative and digital methods. Building on these efforts, incorporating the voices and lived experiences of AYA survivors in the generation and dissemination of health information is critical and has been lacking from published studies.

Today, 9.9 million vaccine doses aimed to target COVID-19 have been administered globally [WHO Coronavirus (COVID-19) Dashboard, 2021]. The mental health status of AYA survivors may be different now than early in the pandemic. However, with the emergence of new SARS-CoV2 variants, coupled with changing public health responses around the world, AYA survivors continue to grapple with a great deal of uncertainty related to their potential risks and management of COVID-19. In particular, the mental health of AYA survivors may be compromised in this quickly evolving context. Therefore, a complete assessment of the impact of COVID-19 on the mental health status of AYA survivors, along with the dissemination of tailored, evidence-based information on coping with mental health during COVID-19 in an accessible manner, is necessary to support the on-going well-being of this at-risk and vulnerable group.

## Current Research

The overarching goal of the current research was to identify the impact of COVID-19 on the mental health status of AYA survivors during May and June 2020 and determine their information needs living in the pandemic, particularly given their prior diagnosis and treatment exposure. This goal was carried out through the following aims:

### Aim 1a

To explore the perceived impact of COVID-19 on the mental health status of AYA survivors, including ratings of anxiety, depression, and PTSS.

### Aim 1b

To determine differences in the mental health status of AYA survivors who reported that their mental health status was associated with fears/worries about their past cancer and treatment, with those who did not report the same.

### Aim 2a

To describe the COVID-19-related information needs of AYA survivors.

### Aim 2b

To disseminate an infographic outlining our research and evidence-based coping strategies for COVID-19 specific to survivors of childhood cancer.

## Materials and Methods

### Patient and Public Involvement

We reported the background, aims, methods, and results of this study based on the checklist from the Guidance for Reporting Involvement of Patient and the Public Short Form (GRIPP2-SF; Staniszewska et al., 2017). Specifically, the COVID-19 and Childhood Cancer study is a patient-oriented research project and thus developed in collaboration with our program’s patient partners to identify priority areas for our research objectives. Patient partners collaborated on the study design, recruitment, data collection, interpretation of results, and knowledge dissemination.

### Participants

Participants were recruited as part of a larger study on COVID-19 and childhood cancer. AYA survivors were identified as individuals who were: (1) diagnosed with cancer under 21 years of age; (2) more than 5 years from diagnosis and/or more than 2 years from cancer treatment completion, consistent with the definition established by Children’s Oncology Group Long-Term Follow-Up guidelines (Childrens Oncology Group, 2018); (3) currently between the ages of 13 and 39 years of age (National Cancer Institution, 2022). No other restrictions were placed on eligibility to maximize the representativeness of the sample and ecological validity of the findings.

### Recruitment

Participants were recruited through a variety of sources, including social media (e.g., Twitter), community organizations (e.g., Kids Cancer Care Foundation, Childhood Cancer Survivor Canada), and convenience sampling through our patient partners. Ethics approval was obtained by the Health Research Ethics Board of Alberta—Cancer Committee (HREBA.CC-20-0151). Data were collected between May and June 2020.

### Procedure

This study employed a quantitative, cross-sectional design. Participants completed a survey with questions pertaining to their cancer history, current mental health status, and their understanding of COVID-19-related information. The survey was administered through REDCap™, a secure online platform affiliated with the tertiary care pediatric hospital where the research was based.

### Measures

#### Mental Health

Standardized measures of anxiety, depression, and PTSS were administered as an index of survivors’ current mental health status. Each measure is described in detail below.

##### Anxiety

Anxiety was measured using the anxiety subscale from the Patient Reported Outcomes Measurement Information System (PROMIS Profile-29; e.g., Cella et al., 2007). The PROMIS Profile-29 assesses anxiety, depression, fatigue, sleep disturbances, peer relationships and cognitive function, pain interference and pain intensity. This measure has been validated in pediatric oncology for 8 to 17 years (e.g., Hinds et al., 2013). Participants were asked to rate 4 items assessing symptoms of anxiety over the past week (e.g., “I felt worried”) on a five-point Likert scale from 1 “*never*” to 5 “*always*.” Scores range from 4 to 20 with higher scores reflecting greater severity of anxiety. Standardized scores were computed by summing the responses scores on all items to generate a total raw score, which was then converted to a *t*-score with a mean (*M*) of 50 and standard deviation (SD) of 10 based on a US general population. T-scores can be interpreted as follows: < 55: *none* to *slight*; 55–59: *mild*; 60–69: *moderate*; 70+: *severe* levels of anxiety (American Psychiatrists Association, 2022). Internal consistency for this sample was good (*α* = 0.89).

##### Depression

As with anxiety, depression was measured by a subscale from the PROMIS Profile-29 (e.g., Cella et al., 2007) as described above. Participants were asked to rate 4 items assessing depression symptoms (e.g., “I felt sad”) in the past week on a five-point Likert scale from 1 “*never*” to 5 “*always*.” Scores range from 4 to 20 with higher scores reflecting greater severity of depression. Standardized scores were computed by summing responses the scores on all items to generate a total raw score, which was then converted to a *t*-score based on the same norm referencing as the anxiety subscale of the PROMIS Profile-29. Score classification (*none to slight, mild, moderate, and severe*) was based on the same criteria as the anxiety subscale. Internal consistency for this sample was excellent (*α* = 0.93).

##### Post-traumatic Stress Symptoms

PTSS were assessed using two measures. The Child Post-Traumatic Stress Disorder Symptom Scale for DSM-5 (CPSS-5—Self Report Version for DSM-5; ISTSS, 2022) was administered to adolescent survivors. The CPSS-5 is a 27-item self-report measure that assesses PTSD symptoms experienced by children ages 8 to 17 years over the past month. Participants are asked to rate the frequency of PTSS experienced using a five-point Likert scale from 0 “*not at all*” to 4 “*6 or more times a week/almost always*” (e.g., “I have bad dreams or nightmares”). The total severity score ranges from 0 to 80 and was computed by summing the ratings of the first 20 items. Higher score reflecting higher severity of PTSS, and a cutoff score of 31 can be used to identify a probable PTSD diagnosis in children (Foa et al., 2018). Separately, seven items assessing impairment of endorsed symptoms on daily functioning are summed to indicate an impairment score that ranges from 9 to 28. Internal consistency of this sample was excellent (*α* = 0.97).

The PTSD Checklist for DSM-5 (PCL-5; Weathers et al., 2013) was administered to young adult survivors. The PCL-5 is a 20-item self-report measure that assesses PTSD symptoms experienced over the past month by adults 18 years and older. Participants are asked to rate the extent to which they are likely to experience PTSS using a five-point Likert scale from 0 “*not at all*” to 4 “*extremely*” (e.g., “I am bothered by repeated, disturbing, and unwanted memories of the stressful experience”). Total score was computed by summing the scores of all items. Scores range from 0 to 80 with higher score reflecting higher severity of PTSS. A cutoff score between 31 and 33 is indicative of a probable diagnosis of PTSD. In this study, we referred to a lower cutoff point to increase detection of possible cases of PTSD. Internal consistency of this sample was excellent (*α* = 0.95).

#### Perceived COVID-19 Impact

Participants responded to 6 questions regarding the perceived impact of COVID-19 on their current mental health status and daily living circumstances. These items were developed by the research team with experts (researchers, health care providers) in pediatric psychosocial oncology and in collaboration with our patient partners to shape research priorities. An example item regarding the perceived impact of COVID-19 on mental health status included: “Is your current mental health tied to fears/worries about… [your] past cancer and treatment?” Participants were asked to endorse “yes” or “no” in response to this item. An example item regarding the perceived impact of COVID-19 on daily living included: “What COVID-19 restrictions are currently in place where you live?” Participants were asked to select all that applied to them from a series of options, such as “school cancelled,” “public gatherings limited to <5 people,” and/or “must wear mask/face covering in public.” See Data Sheet 1 for a full version of this COVID-19 questionnaire.

#### COVID-19-Related Information Needs

Participants were asked to answer 5 questions regarding their COVID-19-related information needs. These items were also developed by the research team and in collaboration with patient partners. Example items included: “Have you received information related to the potential risks of COVID-19 as a survivor of childhood cancer?” and “Would you like to receive more information about your specific risks from COVID-19 as a survivor of childhood cancer?” Participants were also asked to identify specific materials or resources that may help to improve their mental health by selecting all that applied to them from a list of options, such as “information specific to cancer survivors regarding mental health,” “online social connections,” and/or “general information regarding mental health.” See Data Sheet 1 for a full version of this COVID-19 questionnaire.

#### Demographic Information

Participants completed a demographic form. Information regarding their date of birth, sex, gender, ethnicity, and country of residence was collected.

#### Cancer History

Participants answered questions regarding their cancer history, including their age of diagnosis, cancer diagnosis, type of treatment, and years of treatment. Three items adapted from the Self-Report Survey of Cancer Knowledge by Kunin-Batson et al. (2016) were also included. An example item is: “Did your cancer treatment cause any health problems you are currently experiencing?”

### Statistical Analyses

Preliminary screening was conducted to assess for any missing data, outliers, multicollinearity, and normality. All analyses were computed on SPSS 27.0 (IBM Corp, 2020). To address Aim 1, we provided a summary of descriptive statistics of participant responses, including their clinical characteristics and mental health status. Pairwise deletion was used to handle missingness in descriptive analyses. For exploratory purposes, we conducted independent *t*-tests and a one-way ANOVA to explore differences in patient and clinical characteristics in relation to study outcomes. We conducted independent *t*-tests to explore how differences between participants that reported their current mental health status was tied to fears/worries related to their past cancer and treatment, with those who denied the same, in relation to their mental health status, indexed by anxiety, depression, and PTSS. PTSS scores were standardized to combine data collected from CPSS-5 and PCL-5. All *t*-tests were bootstrapped to generate confidence intervals (CIs) and further reduce any effects of possible non-normality or outliers. Where possible, effect sizes (Cohen’s *d;* Cohen, 2013) are reported. To address Aim 2, we provided a summary of descriptive statistics of participant responses on their report of their COVID-19-related information needs. We also developed and disseminated an infographic summarizing preliminary findings and evidence-based, recommended strategies for coping with the pandemic.

## Results

### Participant and Clinical Characteristics

One hundred and six participants originally completed the study questionnaire. However, 12 participants were removed due to ineligibility as per study criteria (8 participants were over 39 years old, 1 participant mis-entered their date of birth, 3 participants did not report their date of birth). As a result, a total of 94 participants were included in the data analysis with a mean age of 26.9 years (SD = 6.2). Of this sample, 79 participants (84.0%) reported that their sex assigned at birth was female, 14 participants reported that sex assigned at birth was male, and 1 participant preferred not to answer. Regarding their gender, 78 participants identified as female, 13 as male, 2 as non-binary, and 1 preferred not to answer. Participants were asked to describe their ethnicity among listed categories and indicate all that applied to them. They most commonly identified as White (*n* = 88; 93.6%), multi-ethnic (*n* = 9; 9.6%), and East Asian (*n* = 4; 4.3%). Participants reported residing in 10 countries, most commonly Canada (*n* = 37; 40.2%), the United States (*n* = 28; 30.4%), and the UK (*n* = 6; 15.2%).

Participants elected to complete questions regarding their cancer history. AYA cancer diagnoses included lymphoma (*n* = 30; 34.1%), leukemia (*n* = 26; 29.5%), solid tumour (*n* = 24; 27.3%), and brain tumours (*n* = 8; 9.1%). The mean age of diagnosis was 11.01 years (*SD* = 5.49) and the mean years off treatment was 14.90 (SD *=* 8.35). The types of treatment received included chemotherapy (*n* = 84; 95.5%), surgery (*n* = 67; 76.1%), radiation (*n* = 32; 36.4%), and bone marrow transplant (*n* = 7; 8.0%). A summary of participant and clinical characteristics is provided in Table 1.

**Table 1:** Participant and clinical characteristics.

	*n* (%)	*M* (SD)
**Participant Demographic (** ***n*** ** = 94)**		
**Age**		26.87 (6.23)
**Sex**		
Female	79 (84.0)	
Male	14 (14.9)	
Other	1 (1.1)	
**Gender**		
Female	78 (83.0)	
Male	13 (13.8)	
Non-binary	2 (2.1)	
Prefer not to answer	1 (1.1)	
**Ethnicity** ^1^		
White	88 (93.6)	
Identified as multi-ethnic	9 (9.6)	
East Asian (e.g., Chinese, Japanese, Korean)	4 (4.3)	
Arab	2 (2.1)	
Black	2 (2.1)	
South Asian (e.g., East Indian, Pakistani, Sri Lankan)	2 (2.1)	
Aboriginal (First Nations, Inuit, Métis)	1 (1.1)	
Latin American	1 (1.1)	
Southeast Asian (e.g., Vietnamese, Cambodian, Thai)	1 (1.1)	
West Asian (e.g., Iranian, Afghan)	1 (1.1)	
Other	1 (1.1)	
Prefer not to answer	1 (1.1)	
**Country of Residence** ^2^		
Canada	37 (40.2)	
USA	28 (30.4)	
England (Identified separately)	8 (8.7)	
Ireland	7 (7.6)	
UK	6 (6.5)	
Austria	2 (2.2)	
Australia	1 (1.1)	
Finland	1 (1.1)	
Germany	1 (1.1)	
Japan	1 (1.1)	
**Clinical Characteristics (** ***n*** ** = 88)**		
**Age of Diagnosis (years)**		11.01 (5.49)
**Cancer Diagnosis**		
Lymphoma (e.g., Hodgkin’s, non-Hodgkin’s)	30 (34.1)	
Leukemia (e.g., ALL, AML)	26 (29.5)	
Solid Tumor (e.g., Wilms’ tumor, osteosarcoma)	24 (27.3)	
Brain Tumor (e.g., Medulloblastoma)	8 (9.1)	
**Treatment Received** ^1^		
Chemotherapy	84 (95.5)	
Surgery	67 (76.1)	
Radiation Therapy	32 (36.4)	
Bone Marrow Transplant	7 (8.0)	
**Years off-treatment**		14.90 (8.35)
***Have you had a relapse or second cancer diagnosis?***		
No	78 (88.6)	
Yes	10 (11.4)	
***Did cancer treatment cause any health problems you are currently experiencing?***		
No	32 (36.4)	
Yes	56 (63.6)	
***Do you have any other health conditions/concerns?***		
No	55 (62.5)	
Yes	33 (37.5)	
***Do you feel that your cancer treatment could cause serious future problems?***		
No	12 (13.6)	
Yes	59 (67.0)	
I do not know	17 (19.3)	

1 Participants were asked to select all that applied. Total number of responses can exceed total number of participants.

2 Two participants did not complete this item (n = 92).

Differences in participant characteristics, including sex and gender, were explored in relation to study outcome variables, including anxiety, depression, and PTSS. No significant differences emerged. Likewise, differences in type of cancer diagnosis in relation to the same study outcome variables were explored, and no significant relations were observed. See Supplementary Table 1 for complete results.

### COVID-19 Characteristics

The most common COVID-19 restrictions reported by participants in their respective place of residence were physical/social distancing in public (*n* = 60; 63.8%), community re-launch plans (*n* = 60; 63.8%) and remote learning for school/education (*n* = 52; 55.3%). Five participants (6%) reported that they were infected with COVID-19. Among these individuals, 4 participants (5%) reported that they have recovered from symptoms of COVID-19. All COVID-19 characteristics are provided in Table 2.

**Table 2:** COVID-19 characteristics.

	*n* (%)
***What COVID-19 restrictions are currently in place where you live?***	
Community beginning to re-open stores and services	60 (63.8)
Must maintain physical or social distance when in public	60 (63.8)
School being offered *via* remote learning	52 (55.3)
School cancelled	40 (42.6)
Must wear mask/face covering in public	29 (30.9)
Public gatherings limited to <5 people	23 (24.5)
Public gatherings limited to <50 people	21 (22.3)
Stay at home order (except for essential work or outings)	18 (19.1)
Public gatherings limited to <15 people	15 (16.0)
Others	7 (7.4)
Curfew	3 (3.2)
No restrictions	3 (3.2)
***Have you been told by a doctor or other health care professional that you have, or have had COVID-19?***	
No	76 (93.8)
Yes, and the condition is no longer present	4 (4.9)
Yes, and the condition is still present	1 (1.2)
***Have you been exposed to someone who has been diagnosed with COVID-19***	
No	74 (91.4)
Yes	7 (8.6)

### Aim 1a: To Explore the Perceived Impact of COVID-19 on the Mental Health Status AYA Survivors, Including Ratings of Anxiety, Depression, and PTSS

**Figure 1:**
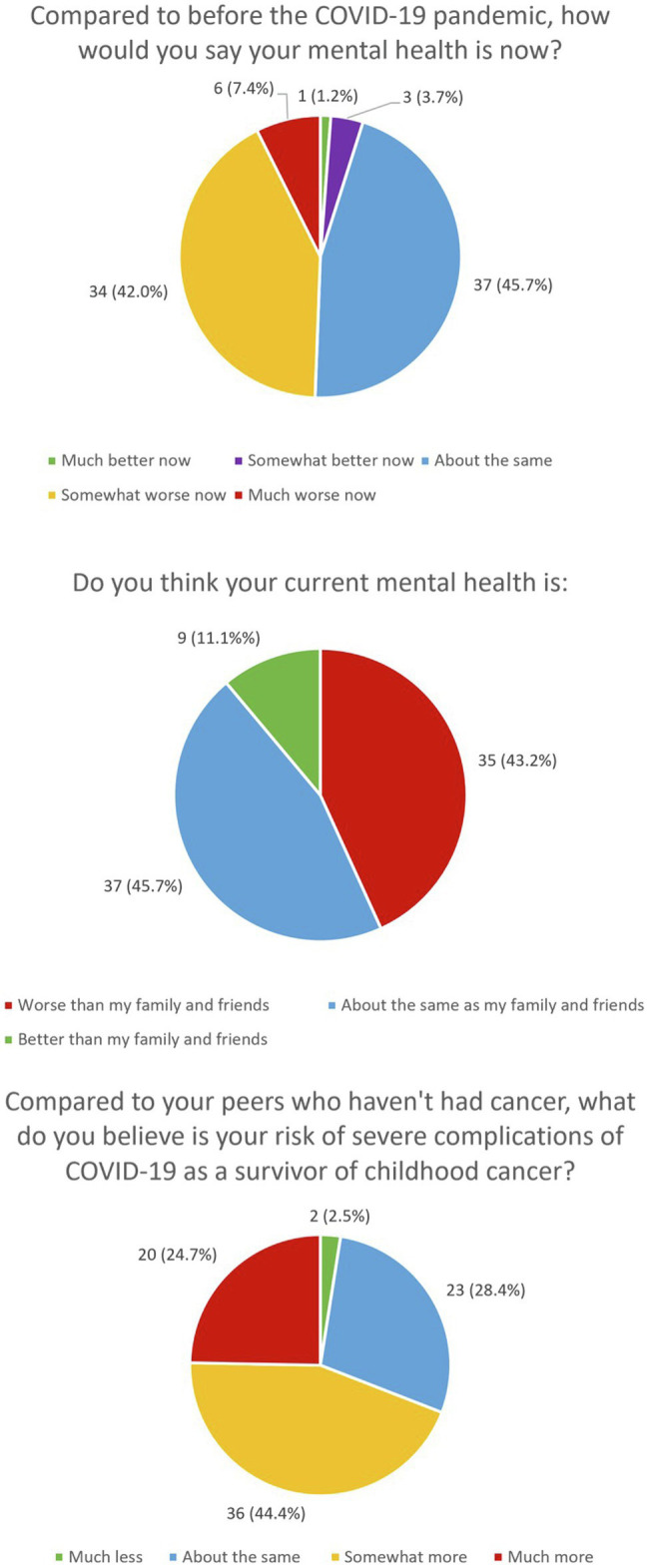
The perceived impact of COVID-19 on the mental health status of AYA survivors.

Participants reported on their perceived risk of severe complications from COVID-19 in comparison to their peers who had not had cancer. More than half of the participants (69.1%) indicated that their risk was *somewhat more* to *much more* than their peers. In addition, 23 participants (28.4%) rated their risk as *about the same* as their peers and 2 participants (2.5%) rated their risk as much less than their peers. These findings are summarized under Figure 1.

Participants described their mental health status now compared to prior to the pandemic. Almost half of the participants (49%) rated their mental health status ranged from *somewhat worse* to *much worse* now, while 37 participants (39.4%) rated that their mental health status as having *stayed about the same*. Four participants (4.9%) reported that their mental health status ranged from *somewhat better* to *much better* now. Participants were also asked to describe how well their mental health fared in comparison with their family and friends, with 35 participants (37.2%) reporting that they were *worse*, 37 participants (39.4%%) reporting that they were *about the same*, and 9 participants (10%) reporting they were *better* than those of their family and friends. These findings are summarized under Figure 1.

Participants further reported on their mental health status using clinical rating scales of anxiety, depression, and PTSS. While participant scores for anxiety were on average in the *mild* range (*M* = 58.8, SD = 9.3), 33 participants (42.3%) reported experiencing *moderate* to *severe* levels of anxiety. Additionally, participant scores for depression were on average in the *mild* range (*M* = 55.4, SD = 10.3), and 22 participants (28.2%) reported experiencing *moderate* to *severe l*evels of depression. Finally, participant scores of PTSS for adolescent survivors that completed the CPSS were on average below the clinical cutoff (*M* = 28.3, SD = 20.6) with one participant (1.1%) reporting clinically significant levels of PTSD symptoms. Scores of PTSS for young adult survivors who completed the PCL-5 were on average below the clinical cutoff (*M* = 22.0, SD = 18.0) with 18 participants (19.1%) indicating clinically significant levels of PTSD symptoms.

Participants identified factors that impacted their mental health status during COVID-19. Notably, 39 participants (41.5%) indicated that their current mental health status was tied to fears/worries about their past cancer. Similarly, 40.4% (*n* = 38) of respondents indicated that their mental health status was related to fears/worries about catching COVID-19. The remainder of participants identified media and messaging about the pandemic (19.1%, *n* = 18), nothing in particular (10.6%, *n* = 10), other (5.3%, *n* = 5; e.g., “no supports available with new baby”), and not applicable (2.1%, *n* = 2) as factors related to their mental health status.

### Aim 1b: To Compare the Mental Health Status of AYA Survivors Who Reported That Their Mental Health Status Was Tied to Fears/Worries About Their Past Cancer and Treatment, With Those Who Did Not Report the Same Experience

Thirty-eight participants who indicated that their current mental health status was tied to fears/worries about their past cancer and treatment, reported significantly higher levels of anxiety (*M* = 62.05, SD = 8.15; *moderate* range) compared to the 40 participants who did not report the same (*M* = 55.71, SD = 9.33; *mild* range; *t*(76) = 3.19, *p* = 0.002, *d = 0*.72, 95% CI [2.38, 10.30]). No significant difference in levels of depression was observed between the 38 participants who shared that their current mental health status was tied to fears/worries about their past cancer history (*M* = 57.17, SD = 10.25; *mild* range) compared to the 40 participants who did not report the same association (*M* = 53.67, SD = 10.25; *none to slight* range; *t*(76) = 1.51, *p* = 0.135, *d = 0*.34, 95% CI [−1.12, 8.13]). Finally, 37 participants who endorsed their current mental health status was tied to fears/worries about their past cancer and treatment (*M =* 0.34, SD *=* 1.07) reported significantly higher levels of PTSS than the 38 participants who did not endorse the same (*M =* −0.33, SD *=* 0.79*, t*(73) = −3.12, *p* = 0.003; *d* = 0.94, 95% CI [−1.11, −0.24]). These results are reported in Table 3.

**Table 3:** Independent *t*-tests comparing the beliefs of AYA survivors of childhood cancer regarding their current mental health in relation to their past cancer treatment and experience (2: fears/worries, no fears/worries) and how this related to their current mental health (3, anxiety, depression, PTSS).

	Fears/Worries			No Fears/Worries								
	*n*	*M*	*SD*	*n*	*M*	*SD*	*t*-test	*df*	*p*	Cohen’s *d*	95%	CI
Anxiety	38	62.05	8.15	40	55.71	9.33	3.19^*^	76	0.002	0.72	2.38	10.30
Depression	38	57.17	10.25	40	53.67	10.25	1.51	76	0.135	0.34	−1.12	8.13
PTSS^†^	37	0.34	1.07	38	−0.33	0.79	−3.12^*^	73	0.003	0.94	−1.11	−0.024

^*^p < 0.001. ^†^PTSS was computed using the CPSS for survivors < 18 years and the PCL-5 for survivors ≥ 18 years. Scores were standardized to compute a combined index of PTSS.

### Aim 2a: To Describe the COVID-19-Related Information Needs of AYA Survivors

Over three-quarters of the participants (76.5%) indicated that they had not received any information related to the potential risks of COVID-19 as a survivor of childhood cancer, while 19.8% reported they had received some information, and 3.7% indicated that they did not know. Of note, 13.8% of participants did not answer this question. Of those who had received information related to potential risks of COVID-19, 18.8% reported it had been *definitely* helpful, 50.0% as *moderately* helpful, and 31.3% as *slightly* helpful. Participants were also asked if they would like to receive more information regarding risks and guidelines and 69.1% endorsed that they would like to receive more information on their specific risks from COVID-19 as a survivor of childhood cancer, while 59.3% of participants wanted more information about guidelines and recommendations for survivors of childhood cancer during the COVID-19 pandemic. These data are presented in Table 4.

**Table 4:** COVID-19-related information needs of AYA survivors.

	*n* (%)
***Have you received information related to the potential risks of COVID-19 as a survivor of childhood cancer***	
No	62 (76.5)
Yes	16 (19.8)
I do not know	3 (3.7)
***Where did you receive this information?***	
My healthcare team	11 (11.7)
Childhood cancer specialist organizations	9 (9.6)
Family and/or friends	2 (2.1)
Mass media	0 (0.0)
Social media	0 (0.0)
Other	0 (0.0)
***This information has been:***	
Definitely helpful	3 (18.8)
Moderately helpful	8 (50.0)
Slightly helpful	5 (31.3)
***Would you like to receive more information about your specific risks from COVID-19 as a survivor of childhood cancer?***	
No	25 (30.9)
Yes	56 (69.1)
***Are there any materials or resources that may help improve your mental health at this time?***	
Information specific to cancer survivors regarding mental health	34 (36.2)
Not applicable	30 (31.9)
Online social connection with other survivors	18 (19.1)
General information regarding mental health	15 (16.0)
Online connection with health-care providers	13 (13.8)
Other	2 (2.1)
***Would you like to receive more information about guidelines and recommendations for survivors of childhood cancer during the COVID-19 pandemic?***
No, I do not need specific recommendations for survivors	33 (40.7)
Yes, I would like specific recommendations	48 (59.3)

### Aim 2b: To Develop and Disseminate an Infographic Detailing Evidence-Based Coping Strategies for COVID-19 Specific to Survivors of Childhood Cancer

**Figure 2:**
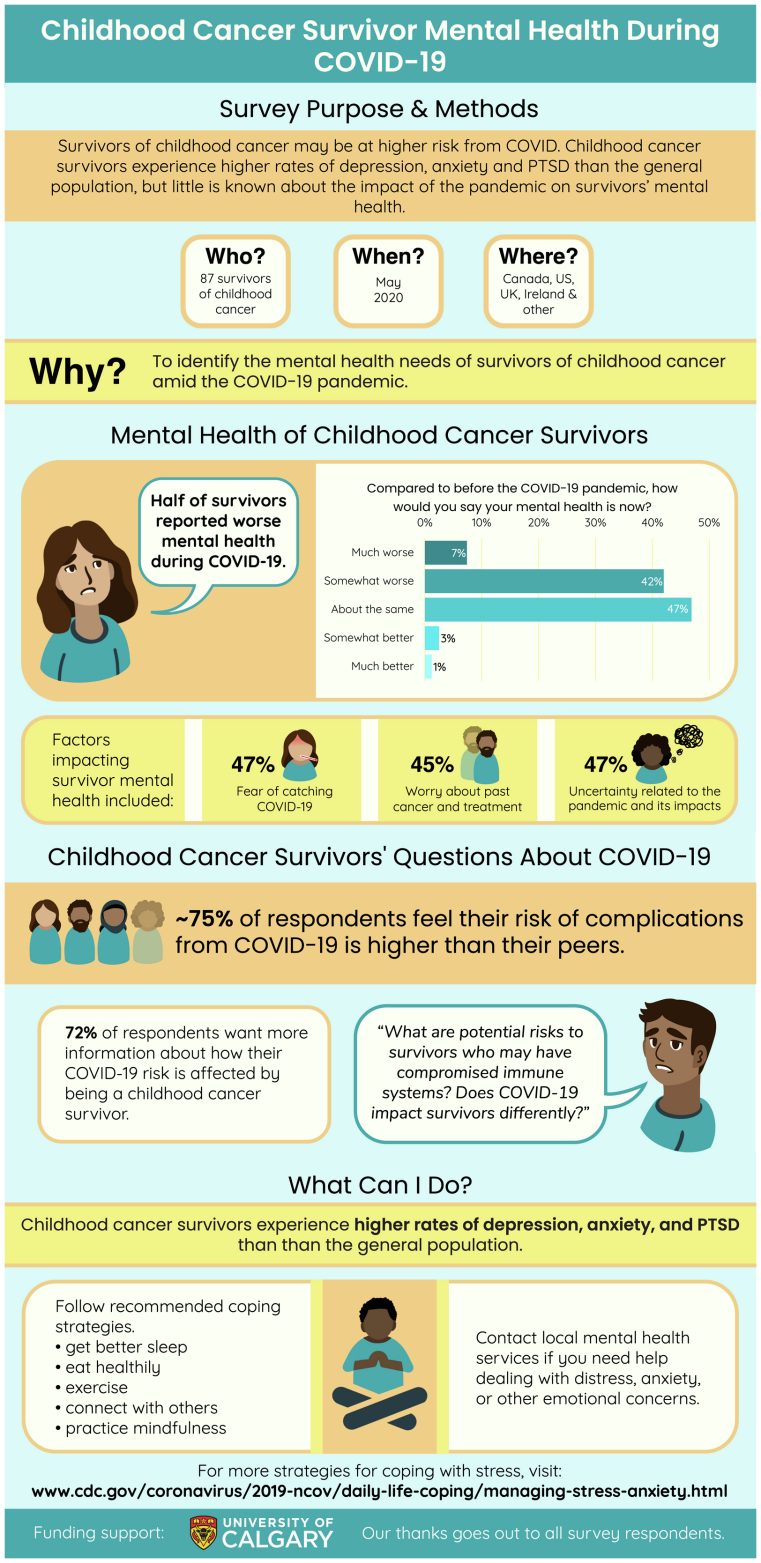
Infographic for AYA survivors of childhood cancer on coping with COVID-19.

Based on the outcomes identified above, our research team developed and disseminated an infographic summarizing preliminary findings from the current study, along with recommended strategies for coping with their mental health in the pandemic. Content development was informed by our research team through expert consultation. Illustrations were created by graphic designers that specialize in scientific illustrations (Scientific Illustrations, 2022). Feedback of initial iterations was gathered from the research team and patient partners. This iterative process resulted in the development of an illustration focused on: (1) the purpose of the research, participant characteristics, timing of the study and study location, as well as the study rationale; (2) the mental health status of survivors, and factors that impacted their mental health; (3) concerns expressed by survivors regarding their health risks from COVID-19 identified through the current study; (4) recommended coping strategies (e.g., diet, sleep, exercise) and access to online (e.g., Center for Disease Control and Prevention; CDCBreastCancer, 2022) and local, Canadian health services for help. The infographic was shared widely through our research team, patient advocacy groups and community organizations (e.g., Kids Cancer Care Foundation), and social media (i.e., Twitter) in March 2021. As of January 2022, a review of Twitter analytics showed a total of 11, 903 impressions, 502 engagements, 56 “likes” and 22 re-shares (or “retweets”) of the infographic disseminated. The infographic is displayed in Figure 2.

## Discussion

This study explored the perceived impact of the COVID-19 pandemic on the mental health status of AYA survivors. Of the 40% of respondents reporting that their mental health status amid the early stages of the COVID-19 pandemic was linked to their fears/worries about their past cancer and treatment, they reported experiencing greater levels of anxiety and PTSS than those that did not report the same. Additionally, AYA survivors perceived themselves to be at a higher risk from COVID-19 than their family and friends. Despite this, less than a quarter of the participants had received information related to their potential risk, and over half of the participants expressed an interest in receiving more health information. This finding led to our development and dissemination of an infographic reporting our research process and evidence-based, tailored recommendations for how AYA survivors can cope with their mental health during the pandemic.

Nearly half of the participants described their mental health status as worse now compared to before the pandemic. These results are concerning as it is well documented that survivors of childhood cancer already are more likely to experience mental health issues compared to their peers that did not have cancer (Forster and Schulte, 2021; National Cancer Institution, 2022). This means that, for many, they may have already been grappling with anxiety, depression, and PTSS prior to the pandemic and that the onset of COVID-19 only added a greater difficulty to their struggles, resulting in worsened mental health. Further, we know that there has been a significant burden of mental health difficulties among young people as a result of the pandemic (Lee et al., 2020; Stroud and Gutman, 2021). Our findings contribute to this literature, showing that many AYA survivors perceived experiencing poor mental health shortly after the onset of the pandemic and highlighting a need to support this already vulnerable population.

Not all AYA survivors experienced poor mental health during the pandemic. In fact, nearly half of the participants described their mental health status as about the same now as compared to before the pandemic. Further, the same proportion of participants did not express a desire for more information on how to cope with their mental health in the pandemic. The lack of an overwhelming report of the negative impact of COVID-19 may reflect a unique advantage for AYA survivors. For some, their cancer experience may serve as a buffer against some of the risks of the pandemic. A qualitative study conducted by Shay et al. (2021) with AYA patients and survivors identified perceived unexpected advantage of a cancer history during the early stages of the global pandemic. Specifically, AYA survivors relied on strategies that they previously used to cope with their cancer and treatment. Many also accessed social support through online cancer-specific networks that they previously established during their cancer experience (Schulte et al., 2020). Other, more recent studies have found a similar sign of resilience and protective mechanisms identified in survivors and their families as they navigate the pandemic (e.g., Wimberly et al., 2021; Jacobson et al., 2022). In effect, not all AYA survivors are struggling with their mental health due to their past cancer and treatment. Rather, there may be existing areas of strength and/or skills that AYA survivors can leverage for their coping to help alleviate or buffer some of the consequences of living with COVID-19. These discoveries can inform future research priorities on developing interventions for AYA survivors in coping with their mental health during this on-going pandemic.

AYA survivors who reported that their mental health status was tied to fears/worries about their past cancer and treatment reported significantly worse anxiety and PTSS than those who did not report the same. This, along with an expressed interest in receiving more health information, suggests that AYA survivors may benefit from learning more about their potential COVID-19-related health risks and the way in which this information is delivered should account for these perceived mental health vulnerabilities. However, there appears to be a knowledge gap among survivors of childhood cancer in receiving information related to their specific risks both before and during the pandemic. In fact, only 35% of survivors recognize that they could develop a serious health problem as a result of their cancer history (Kadan-Lottick et al., 2002). As health care providers and researchers, it is a priority that we find better ways to share knowledge with this population.

This study provided the basis for the development and dissemination of an infographic as a means to share health information with AYA survivors, including evidence-based recommendations for mental health coping strategies. The creation of this infographic in collaboration with our patient partners was intended to ensure the engagement and input of those with lived experience as an AYA survivor of pediatric cancer in the research process (Staniszewska et al., 2017). We communicated the nature of our research project to AYA survivors with the goal of increasing transparency and accessibility of our work. Further, in the context of a quickly changing global pandemic, we produced this infographic in an effort to quickly respond to the acute needs of AYA survivors early in the pandemic and in turn support their on-going coping with their mental health and overall quality of life.

It is worth noting that current methods of disseminating health information to survivors (e.g., survivorship care plans) are not always effective (Jacobsen et al., 2018). A recent meta-analysis showed that survivorship care plans do not improve patient-reported outcomes, including anxiety, depression, or other cancer-related distress (Hill et al., 2020). Likely, a multi-pronged approach is required to reach survivors, partnering with patients on how and what to distribute are important considerations. There are studies that show AYA survivors need a better way to learn about and engage in their own health information, including meeting them where they are, increased accessibility, and greater equity in receipt of care (Oeffinger et al., 1998; Mertens et al., 2004; Taylor et al., 2004). The development of innovative, childhood cancer-specific, and personalized interventions are just beginning (Devine et al., 2018; McLoone et al., 2020). Our development of an infographic is a unique contribution to this line of inquiry, and future research is needed to evaluate the effectiveness of disseminating these knowledge translation tools, as well as the feasibility of implementation.

There are several limitations to be addressed in future research. The effects of the COVID-19 pandemic are inherently complex. We only examined the mental health status of AYA survivors, as indexed by anxiety, depression, and PTSS at the individual level. Other, multi-level factors were not accounted for. For example, due to insufficient sample size, the current research does not account for group (e.g., country) level analysis that may underlie the current findings (e.g., Lai et al., 2020). Additional studies that incorporate multi-level techniques with adequate sampling distribution may help to clarify some of the influences at the societal and global level on the well-being of AYA survivors. Further, we relied on self-report from AYA survivors regarding their perception of their potential risks to COVID-19. We did not capture open-ended responses to seek specific reasons or motivations behind AYA survivors’ perception on this matter. Future research capturing these insights through open-ended response format or qualitative interventions may help to contextualize the experiences of AYA survivors. Our cross-sectional assessment of AYA survivors only allowed us to examine the study participants at one point in time. This did not allow us to capture the possible impact of distinct COVID-19 waves on the experiences of AYA survivors over time. These potential impacts of COVID-19 are dynamic and chronic, as are the needs of AYA survivors. Future studies would benefit from a longitudinal evaluation of the experiences of AYA survivors over time in order to further examine the interactional nature of their experiences coupled with their development level, as well as with the pandemic across time. Inclusion of a data aggregation tool on the COVID-19 trajectory may help to address these possibilities and capture changes over time. In addition, there was potentially a sampling bias inherent in our recruitment strategy that may have hindered our efforts in ensuring a representative and diverse sample of participants. Moreover, there was a lack of representation in our sample of AYA survivors collected. This limited our ability to generalize findings from the current research to diverse groups. Research suggests that individuals who face systematic health and social inequities are at a greater risk of getting sick and dying (Dalton et al., 2008; Bambra et al., 2010; Devine et al., 2018). Therefore, future research ensuring a representative group, such as an inclusion of those who are non-English speaking, from different migration status, and/or those from rural geographic regions, will be essential to ensuring we are capturing the lived experiences of all individuals.

## Conclusion

COVID-19 is an on-going and global pandemic with serious implications on the mental health of all individuals. As an at-risk group, AYA survivors may be especially vulnerable to the psychosocial impact of the COVID-19 pandemic. We found that in the early pandemic, between May and June 2020, AYA survivors perceived that their fears and worries about their past cancer and treatment contributed to their current mental health status (anxiety, PTSS). AYA survivors also identified a desire for COVID-19-related information. We therefore developed an infographic to help AYA survivors to have better access to practical health information to support their coping and quality of living. With the continued emergence of new SARS-CoV2 variants coupled with the widespread distribution of COVID-19 vaccines and treatments, future research focused on a comprehensive and longitudinal assessment of the mental health status of AYA survivors will be helpful to determine ways that we can continue to support the psychosocial needs of this vulnerable population during this unprecedented crisis.

## Data Availability Statement

The raw data supporting the conclusions of this article will be made available by the authors, without undue reservation.

## Ethics Statement

The studies involving human participants were reviewed and approved by Health Research Ethics Board of Alberta – Cancer Committee (HREBA.CC-20-0151). Written informed consent to participate in this study was provided by the participants’ legal guardian/next of kin.

## Author Contributions

FS contributed to study conceptualization, methods, data collection, knowledge translation/dissemination (infographic), and writing (preparing the original draft and reviewing and editing). SH contributed to conducting data analysis and writing (preparing the original draft and reviewing and editing). CF, EA, and MS contributed to study development and data collection. AT and SC contributed to data analysis and writing (preparing the original draft and reviewing and editing). VF, CW, LW, LH, GM, PP, and KR contributed to study conceptualization, knowledge translation/dissemination (infographic, review, and feedback), and writing (reviewing and editing). All authors contributed to the article and approved the submitted version.

## Funding

CW is supported by the National Health and Medical Research Council of Australia (APP1143767 and APP2008300). SH is supported by the Cumming School of Medicine Postdoctoral Scholarship, and the Arnie Charbonneau Cancer Institute. FS is supported by Alberta Children’s Hospital Research Institute, Charbonneau Cancer, Research Institute, and the Button Family Initiative.

## Conflict of Interest

The authors declare that the research was conducted in the absence of any commercial or financial relationships that could be construed as a potential conflict of interest.

## Publisher’s Note

All claims expressed in this article are solely those of the authors and do not necessarily represent those of their affiliated organizations, or those of the publisher, the editors and the reviewers. Any product that may be evaluated in this article, or claim that may be made by its manufacturer, is not guaranteed or endorsed by the publisher.
