# Supplementary material for: The Perceived Impact of COVID-19 on the Mental Health Status of Adolescent and Young Adult Survivors of Childhood Cancer and the Development of a Knowledge Translation Tool to Support Their Information Needs
Source: Front Psychol. 2022 May 30;13:867151. doi: 10.3389/fpsyg.2022.867151 (PMC9285488; doi:10.3389/fpsyg.2022.867151)
Supplement: Supplementary file 1 [file Data_Sheet_1.pdf]

COVID-19 Study Questionnaire

1. What COVID-19 restrictions are currently in place where your child lives? (Check all that apply)	- Schools cancelled<br>- School being offered via remote learning<br>- Public gatherings <5 people<br>- Public gatherings <15 people<br>- Public gatherings <50 people<br>- Must wear mask/face covering in public<br>- Must maintain physical or social distance when in public<br>- Curfew<br>- Stay at home order (except for essential work or outings)<br>- Community relaunch plans<br>- No restrictions<br>- Other (please specify) __________________
2. Have you been told by a doctor or other health care professional that you have, or have had COVID-19 (novel coronavirus)?	- No<br>- Yes, and the condition is still present<br>- Yes, and the condition is no longer present
3. Have you been exposed to someone who has been diagnosed with COVID-19?	- No<br>- Yes
4. Compared to before the COVID-19 pandemic, how would you say your mental health is **now**?	- Much better now<br>- Somewhat better now<br>- About the same<br>- Somewhat worse now<br>- Much worse now
5. Do you think your current mental health is:	- About the same as my family and friends<br>- Better than my family and friends<br>- Worse than my family and friends
6. Compared to your peers who haven't had cancer, what do you believe is your risk of severe complications of COVID-19 as a survivor of childhood cancer?	- 1, Much less than your peers<br>- 2, Somewhat less than your peers<br>- 3, About the same as your peers<br>- 4, Somewhat more than t your peers<br>- 5, Much more than your peers
7. Is your current mental health:	- Tied to fears/worries about my past cancer and treatment<br>- Tied to the media and messaging about the COVID-19 pandemic<br>- Tied to the media and messaging about the COVID-19 pandemic<br>- Tied to fears/worries about the uncertainty related to the COVID-19 pandemic<br>- Tied to fears/worries about the uncertainty related to how COVID-19 might impact me as a survivor of childhood cancer<br>- Tied to fears/worries about other factors in my life<br>- Not tied to anything in particular<br>- Other (Please specify)<br>- Not applicable
8. Have you received information related to the potential risks of COVID-19 as a survivor of childhood cancer?	- No<br>- Yes<br>- I don't know
9. If yes, where? (Check all that apply)	- Mass media<br>- Social networks<br>- My healthcare team<br>- Family and/or friends<br>- Childhood cancer specialist organizations<br>- Other (please specify)

10. Would you like to receive more information about your specific risks from COVID-19 as a survivor of childhood cancer?	- No, I do not need more specific information as a survivor<br>- Yes, I would like more information
11. Would you like to receive more information about guidelines and recommendations for survivors of childhood cancer during the COVID-19 pandemic?	- No, I do not need specific recommendations as a survivor<br>- Yes, I would like specific recommendations
12. Are there any materials or resources that may help improve your mental health at this time? (check all that apply):	- General information regarding mental health during the COVID-19 pandemic<br>- Information specific to cancer survivors regarding mental health during the COVID-19 pandemic<br>- Online social connection with other survivors<br>- Online connection with health-care providers<br>- Other (please specify)<br>- Not applicable
