# Supplementary material for: The Perceived Impact of COVID-19 on the Mental Health Status of Adolescent and Young Adult Survivors of Childhood Cancer and the Development of a Knowledge Translation Tool to Support Their Information Needs
Source: Front Psychol. 2022 May 30;13:867151. doi: 10.3389/fpsyg.2022.867151 (PMC9285488; doi:10.3389/fpsyg.2022.867151)
Supplement: Supplementary file 2 [file Table_1.DOCX]

**Supplementary Table**

| \| Table S1.  *Exploratory analysis comparing patient (sex, gender) and clinical characteristics (type of cancer diagnosis) with their current mental health status (3: anxiety, depression, PTSS).* \| \| \| \| \| \| \| \| \| \| \| --- \| --- \| --- \| --- \| --- \| --- \| --- \| --- \| --- \| --- \| \| **Participant Characteristics** \| \| \| \| \| \| \| \| \|  \| \| \|  \|  \| ***n*** \| ***M*** \| ***SD*** \| ***t*-test** \| ***df*** \| ***p*** \| **95% CI** \| \| \| \| Anxiety \| Sex \|  \|  \|  \| -0.55 \| 75 \| .586 \| -7.67 \| 4.36 \| \| \|  \| Female \| 66 \| 58.87 \| 9.11 \|  \|  \|  \|  \|  \| \| \|  \| Male \| 11 \| 57.22 \| 10.32 \|  \|  \|  \|  \|  \| \| \|  \| Gender \|  \|  \|  \| -0.59 \| 73 \| .559 \| -8.24 \| 4.49 \| \| \|  \| Female \| 65 \| 58.86 \| 9.18 \|  \|  \|  \|  \|  \| \| \|  \| Male \| 10 \| 56.99 \| 10.84 \|  \|  \|  \|  \|  \| \| \| Depression \| Sex \|  \|  \|  \| 0.51 \| 75 \| .609 \| -4.97 \| 8.43 \| \| \|  \| Female \| 66 \| 54.95 \| 9.74 \|  \|  \|  \|  \|  \| \| \|  \| Male \| 11 \| 56.67 \| 13.53 \|  \|  \|  \|  \|  \| \| \|  \| Gender \|  \|  \|  \| 0.40 \| 73 \| .691 \| -5.58 \| 8.37 \| \| \|  \| Female \| 65 \| 54.72 \| 9.65 \|  \|  \|  \|  \|  \| \| \|  \| Male \| 10 \| 56.12 \| 14.13 \|  \|  \|  \|  \|  \| \| \| PTSS \| Sex \|  \|  \|  \| -0.81 \| 72 \| .423 \| -17.17 \| 7.29 \| \| \|  \| Female \| 64 \| 22.64 \| 18.69 \|  \|  \|  \|  \|  \| \| \|  \| Male \| 10 \| 17.70 \| 12.62 \|  \|  \|  \|  \|  \| \| \|  \| Gender \|  \|  \|  \| -1.11 \| 70 \| .270 \| 19.83 \| 5.64 \| \| \|  \| Female \| 63 \| 22.32 \| 18.66 \|  \|  \|  \|  \|  \| \| \|  \| Male \| 9 \| 15.22 \| 10.50 \|  \|  \|  \|  \|  \| \|   **Clinical Characteristics** | | | | | | |
| --- | --- | --- | --- | --- | --- | --- | --- | --- | --- | --- | --- | --- | --- | --- | --- | --- | --- | --- | --- | --- | --- | --- | --- | --- | --- | --- | --- | --- | --- | --- | --- | --- | --- | --- | --- | --- | --- | --- | --- | --- | --- | --- | --- | --- | --- | --- | --- | --- | --- | --- | --- | --- | --- | --- | --- | --- | --- | --- | --- | --- | --- | --- | --- | --- | --- | --- | --- | --- | --- | --- | --- | --- | --- | --- | --- | --- | --- | --- | --- | --- | --- | --- | --- | --- | --- | --- | --- | --- | --- | --- | --- | --- | --- | --- | --- | --- | --- | --- | --- | --- | --- | --- | --- | --- | --- | --- | --- | --- | --- | --- | --- | --- | --- | --- | --- | --- | --- | --- | --- | --- | --- | --- | --- | --- | --- | --- | --- | --- | --- | --- | --- | --- | --- | --- | --- | --- | --- | --- | --- | --- | --- | --- | --- | --- | --- | --- | --- | --- | --- | --- | --- | --- | --- | --- | --- | --- | --- | --- | --- | --- | --- | --- | --- | --- | --- | --- | --- | --- | --- | --- | --- | --- | --- | --- | --- | --- | --- | --- | --- | --- | --- | --- | --- | --- | --- | --- | --- | --- | --- | --- | --- | --- | --- | --- | --- | --- | --- | --- | --- | --- | --- | --- | --- | --- | --- | --- | --- | --- | --- | --- | --- | --- | --- | --- | --- | --- | --- | --- | --- | --- | --- | --- | --- | --- | --- | --- | --- | --- | --- | --- | --- | --- | --- | --- | --- | --- |
| **Predictor: Type of Cancer Diagnosis** | **Sum of Squares** | ***df*** | **Mean Square** | **F** | ***p*** |  |
| Anxiety |  |  |  |  |  |  |
| Between Groups | 18.65 | 17 | 1.10 | 0.74 | .755 |  |
| Within Groups | 89.57 | 60 | 1.49 |  |  |  |
| Total | 108.22 | 77 |  |  |  |  |
| Depression |  |  |  |  |  |  |
| Between Groups | 31.67 | 18 | 1.76 | 1.36 | .189 |  |
| Within Groups | 76.54 | 59 | 1.30 |  |  |  |
| Total | 108.22 | 77 |  |  |  |  |
